# 720 Transcultural Collaboration in Burn Mass Casualty Response Under Austere Conditions

**DOI:** 10.1093/jbcr/irad045.195

**Published:** 2023-05-15

**Authors:** Richard Arbour

**Affiliations:** Temple University Hospital/Temple University Health System, Philadelphia, Pennsylvania

## Abstract

**Introduction:**

Burn mass-casualty incidents (BMCI) overwhelm local resources challenging communication, triage, basic care provision and education of non-burn specialist personnel. This is more acute in austere conditions such as the recent international BMCI in November, 2021, in an extremely resource-limited developing country. Greater than 250 severely burned survivors overwhelmed local resources requiring an international response. Effective burn care dictated adapting to austere conditions, educating local providers and cultural competence, vital to teaching and obtaining feedback in ways meaningful to host providers and building burn care capacity.

**Methods:**

Needs assessment showed local healthcare providers needed burn care education focused on wound care, fluid management, pain management and assessment. Cultural competence/cultural humility were integral in avoiding ethnocentrism and collaboration barriers. Understanding local language and communication styles (including nonverbal cues) was prioritized. Pre-test/post-test measured baseline burn care knowledge vs. education efficacy. Lecture/discussion, case study, clinical application and class participation were used to disseminate content. Attention was paid to pace of content discussion and nonverbal indications of understanding as English was not the learners first language. Pacing/terminology was adjusted to optimize understanding. Meaningful recognition was accomplished by public congratulations and formal completion certificates.

**Results:**

Pre-test: Number of students-57; high score-80%; low score-27.5%; average score-53.9%. Content was presented as described in methods section and supplemented with bedside instruction. Post-test: Number of students-38; high score-95%; low score-55%; average score-79.3%. Almost immediately, many participants framed and posted their completion certificates and quickly utilized education content when delivering care.

**Conclusions:**

Optimal interdisciplinary/international collaboration for burn care and education in austere conditions mandates cultural humility and a spirit of inquiry on the part of international guest providers to best understand cultural norms in host countries. A spirit of inquiry on local communication and cultural nuances is integral to optimal collaboration, education and building effective local burn care capacity.

**Applicability of Research to Practice:**

When providing care internationally in austere settings, proactive cultural humility, understanding local cultural and communications nuances and developing education/content delivery that can be readily utilized in addressing critical needs is essential. Meaningful recognition for healthcare team members in host countries develops good will and helps build capacity to continue providing health care upon conclusion of the immediate crisis.

